# Atrazine Triggers DNA Damage Response and Induces DNA Double-Strand Breaks in MCF-10A Cells

**DOI:** 10.3390/ijms160714353

**Published:** 2015-06-24

**Authors:** Peixin Huang, John Yang, Jie Ning, Michael Wang, Qisheng Song

**Affiliations:** 1 Department of Agriculture & Environmental Sciences, Lincoln University of Missouri, Jefferson City, MO 65102, USA; 2 Department of Pediatrics, Children’s Mercy Hospitals and Clinics, University of Missouri Kansas City School of Medicine, Kansas City, MO 64108, USA; E-Mail: phuang@cmh.edu; 3 Department of Pathology and Anatomical Sciences, University of Missouri, Columbia, MO 65211, USA; E-Mails: jning@genome.wustl.edu (J.N.); wangmx@missouri.edu (M.W.); 4 Division of Plant Sciences, University of Missouri, Columbia, MO 65211, USA

**Keywords:** atrazine, DNA double-strand breaks, DNA damage response, MCF-10A cells

## Abstract

Atrazine, a pre-emergent herbicide in the chloro-*s*-triazine family, has been widely used in crop lands and often detected in agriculture watersheds, which is considered as a potential threat to human health. Although atrazine and its metabolites showed an elevated incidence of mammary tumors in female Sprague–Dawley (SD) rats, no molecular evidence was found relevant to its carcinogenesis in humans. This study aims to determine whether atrazine could induce the expression of DNA damage response-related proteins in normal human breast epithelial cells (MCF-10A) and to examine the cytotoxicity of atrazine at a molecular level. Our results indicate that a short-term exposure of MCF-10A to an environmentally-detectable concentration of atrazine (0.1 µg/mL) significantly increased the expression of tumor necrosis factor receptor-1 (TNFR1) and phosphorylated Rad17 in the cells. Atrazine treatment increased H2AX phosphorylation (γH2AX) and the formation of γH2AX foci in the nuclei of MCF-10A cells. Atrazine also sequentially elevated DNA damage checkpoint proteins of ATM- and RAD3-related (ATR), ATRIP and phospho-Chk1, suggesting that atrazine could induce DNA double-strand breaks and trigger the DNA damage response ATR-Chk1 pathway in MCF-10A cells. Further investigations are needed to determine whether atrazine-triggered DNA double-strand breaks and DNA damage response ATR-Chk1 pathway occur *in vivo*.

## 1. Introduction

Atrazine, one of the 2-chloro-*s*-triazine herbicides, is a most-widely used pre- and early-post-emergence pesticide for crop production in the world [1]. According to the U.S. Environmental Protection Agency (EPA), more than 34 million kg of atrazine are applied each year in the United States alone. Atrazine is a moderately persistent chemical in the environment and can be often detected in surface and groundwater with a concentration ranging from 20 to 700 μg/L [2]. Although the EPA monitors and enforces a maximum contaminant level (MCL) of atrazine at 3.0 µg/L in public drinking water (Safe Drinking Water Act, 1991), a recent United States Department of Agriculture (USDA) study revealed that the annual mean concentration of atrazine had exceeded the MCL in public drinking water sources, and the concentration in groundwater could be as high as 65 μg/L [3].

Atrazine pollution in surface and ground water is a growing health concern. It is generally considered as an endocrine-disrupting compound (EDC), with adverse effects on the central nervous system [4,5,6], endocrine system [7,8,9] and immune system [10,11]. Even though the classification of atrazine as an EDC has been debated [12], a number of adverse consequences on living organisms, including mammal species, have been reported, especially on the reproductive system of rats [8], pigs [13], fish [14] and amphibians [9,15,16]. However, the impact of atrazine on humans is not fully understood.

Atrazine has been reported to cause an earlier onset and increased incidence of mammary gland tumors in female Sprague–Dawley rat [17], which implies that atrazine may be carcinogenic. Case-control studies showed weak associations of atrazine with non-Hodgkin lymphoma [18,19], increasing the risk of ovarian [20] and prostate cancers [21]. Gammon *et al.* [22] suggested that atrazine causes mammary tumors in rats by affecting the hypothalamus, which consequently influences the pituitary gland and ultimately disrupts luteinizing hormone cycling, leading to increasing endogenous estrogen and prolactin. In rats, this altered exposure of reproductive hormones could accelerate reproductive aging and a hormonal environment conducive to mammary tumor development. However, this mechanism appears not to work in humans, because women who undergo reproductive senescence have low levels of estrogen and prolactin. Several studies indicated that atrazine may cause carcinogenesis by damaging the integrity of DNA and the stability of the cell genome using the comet assay and chromosomal aberration analysis [23,24,25,26]. However, other studies also suggested that the genotoxic effect by atrazine was minimal, if any [27,28,29,30]. Based on the available animal and human data, the International Agency for Research on Cancer (1999) and the U.S. EPA (U.S. EPA Interim Registration Eligibility Decision for Atrazine, 2003) characterized atrazine as “not likely to be carcinogenic in humans”.

Early precancerous lesions in patient tissues, as well as specific oncogene activation in different tumor models have been linked to DNA double-strand breaks (DSBs) and the activation of DNA-damage checkpoints [31]. In response to DNA damage, phosphatidylinositol (PI)-3 kinase-related kinases ATM (ataxia telangiectasia mutated) and ATR (ATM- and Rad3-related) are initially activated and subsequently phosphorylate a number of proteins, including Rad17 and the Chk1 kinase. This proceeds to phosphorylating a variety of proteins that regulate the DNA-damage response (DDR), including cell cycle arrest, stabilization of stalled replication forks and DNA repair [32]. The ATR-Chk1 axis is central to the DDR and critical for maintaining genome integrity, and they are considered as DNA damage sensor proteins in cells. Thus, the ATR-Chk1 axis can be used to test environmental compounds that could induce DNA damage.

In an effort to examine the cytotoxicity of atrazine and the possibility of atrazine-triggered DNA damage and DDR in human cells, human breast epithelial MCF-10A cells were selected as a study model in this study, because atrazine was suggested to increase the incidence of breast cancer in female Sprague–Dawley rats [17]. In addition, MCF-10A cells are non-tumorigenic and considered to be “normal” breast epithelial cells.

## 2. Results and Discussion

### 2.1. Reduction of Cell Viability

**Figure 1:**
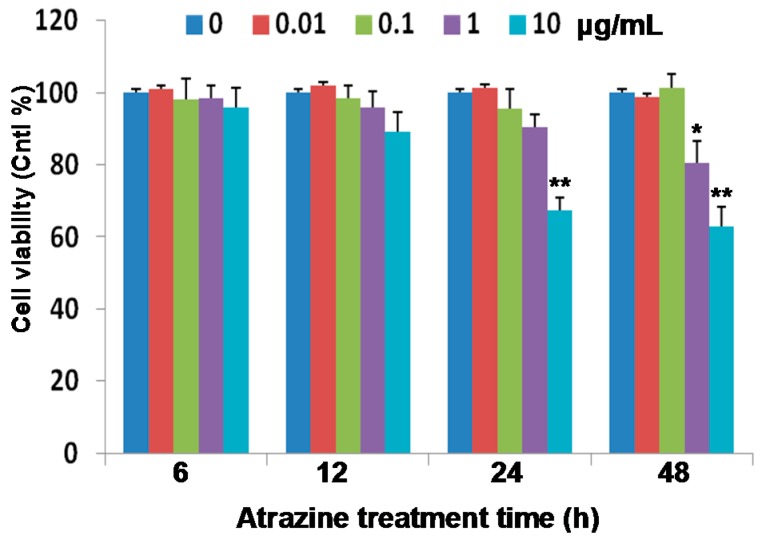
Effects of atrazine on the viability of MCF-10A breast epithelial cells. Cells (1.0 × 10^4^) were incubated for 6, 12, 24 and 48 h in the presence of the indicated concentrations of atrazine or the vehicle (DMSO) control. Cell viability was assayed using an MTS proliferation assay kit. Results are presented as viabilities relative to controls. Experiments were repeated three times (*n* = 4). Data represent the mean ± SD. * *p* < 0.05; ** *p* < 0.01 *vs.* controls.

MCF-10A cells were treated with 0.01, 0.1, 1.0 and 10 µg/mL concentrations of atrazine, for 6, 12, 24 and 48 h, respectively. Cell viability was measured using the MTS [3-(4,5-dimethylthiazol-2-yl)-5-(3-carboxymethoxyphenyl)-2-(4-sulfophenyl)-2H-tetrazolium, inner salt] cell proliferation assay kit. Measurements presented in Figure 1 indicate that, at concentrations of 0.01 and 0.1 µg/mL, atrazine showed no significant adverse impact on the viability of MCF-10A cells. However, at 1.0 µg/mL or higher, the viable cells significantly decreased after a 48 h treatment (*p* < 0.05). At a 10 μg/mL concentration, cell viability dropped after 24 and 48 h of treatment, obviously (*p* < 0.01). Viable cells were decreased to 64% and 61% of controls after treatment with 10 µg/mL of atrazine for 24 and 48 h, respectively.

Atrazine was observed to reduce the cell viability at the concentration of 1.0 to 10 µg/mL for 48 h in MCF-10A cells (Figure 1), possibly by the mechanism of apoptosis. However, it is hard to find a concentration as high as 1.0 to 10 µg/mL of atrazine in the environment; thus, rather than investigating the atrazine-induced cell death, we focused on the early molecular events (e.g., at 6 h) in human MCF-10A cells exposed to the environmentally-detectable level (e.g., 0.1 μg/mL) in our following experiments.

### 2.2. Regulation of Apoptosis-Related Protein Expression

**Figure 2:**
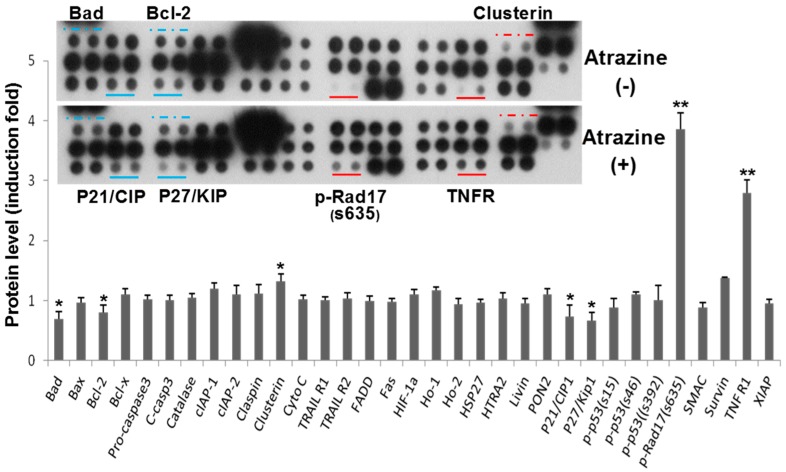
Atrazine regulates proteins related to apoptosis in MCF-10A cells. Cells (1 × 10^6^) were exposed to 0.1 μg/mL of atrazine for 6 h. Cell lysates were used for antibody array analysis, and the top figure shows the representative images of the array. The modulated protein spots in duplicate are designated under the solid lines or above the dashed lines (blue shows downregulated, red shows upregulated protein spots). Data are presented as the mean ± SD of duplicates in fold relative to controls (bottom) using the pixel density scan of the blots (*n* = 4, * *p* < 0.05, ** *p* < 0.01 *vs.* controls).

We then performed apoptosis protein array analysis. The cells were treated with 0.1 µg/mL of atrazine for 6 h, and then, the relative levels of 35 apoptosis-related proteins were measured. Results showed that several apoptotic signaling pathway proteins were modulated following the 0.1 µg/mL of atrazine treatment for 6 h. Pro-apoptotic proteins, including phospho-Rad17 (Ser635) and TNFR1, increased, while Bad, p21 and p27 were inhibited (Figure 2, top). On the other hand, the anti-apoptotic protein clusterin was overexpressed, whereas Bcl-2 decreased.

Pixel density analysis (Figure 2, bottom) indicated that, among the atrazine-affected proteins, phospho-Rad17 (Ser635) increased by 4.1-fold and the death receptor TNFR1 by 2.8-fold, as compared with the untreated control. Among the proteins modulated by atrazine, p-Rad17 (Ser635), TNFR1 and clusterin were significantly elevated, while Bad, Bcl-2, p21 and p27 were inhibited (Figure 2). P-Rad17 (Ser635), p21 and p27 are responsible for regulating the cell cycle, and TNFR1 is a death receptor, while Bad and Bcl-2 modulate the mitochondrial-mediated apoptosis pathway.

### 2.3. Confirmation of Phospho-Rad17 (Ser635) and TNFR1

**Figure 3:**
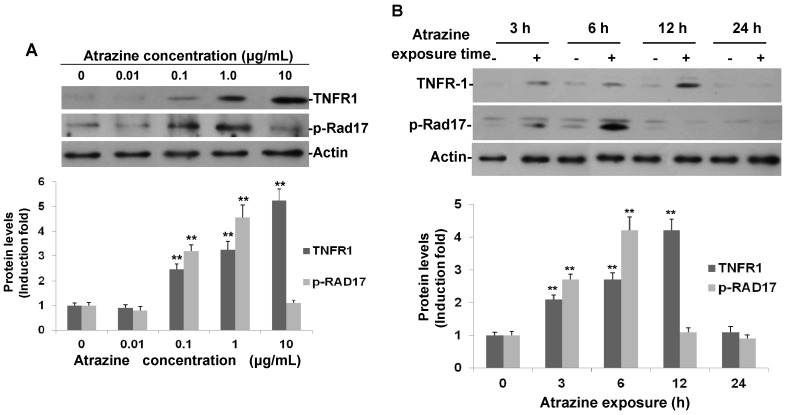
Atrazine induced phospho-Rad17 (Ser635) and TNFR1expression in MCF-10A cells. (**A**) Dose-response of atrazine-induced p-Rad17 (Ser635) and TNFR1. MCF-10A cells (3 × 10^5^) were incubated with indicated concentrations of atrazine for 6 h and analyzed by Western blot; (**B**) Time course of atrazine-induced p-Rad17 (Ser635) and TNFR1 expression in MCF-10A cells. MCF-10A cells (3 × 10^5^) were treated with 0.1 μg/mL of atrazine for the indicated times and analyzed by Western blot. For each blot, the resulting fold-change of atrazine-mediated p-Rad17 and TNFR1 expression over untreated controls is provided in the corresponding histogram (*n* = 3) normalized to actin, ** *p* < 0.01 *vs.* controls.

To verify the involvement of enhanced p-Rad17 and TNFR, which are the most regulated proteins (Figure 2) in response to atrazine in MCF-10A cells, Western blot analysis was performed. The Western blotting results (Figure 3) further validated our findings in the protein array analysis. Both dose- and time-response experiments showed that expression of p-Rad17 and TNFR1 was upregulated by atrazine greatly (Figure 3A,B). At a concentration of 0.01 µg/mL for 6 h, the induction of both proteins was not noticeable. However, at 0.1 and 1.0 µg/mL, phosphorylated Rad17 (Ser635) increased to 3.4- and 4.6-fold of controls, respectively (Figure 3A). However, at a concentration of 10 µg/mL, atrazine-induced p-Rad17 decreased to 1.1-fold of controls. Atrazine significantly induced TNFR1 expression, too (Figure 3A). Unlike p-Rad17, atrazine-induced TNFR1 reached the highest peak at a concentration of 10 µg/mL (Figure 3A). As 0.1 µg/mL of atrazine greatly induced p-RAD17 and TNFR1, we used this concentration for time course experiments (Figure 3B). Our results showed that the induction of p-Rad17 and TNFR1 occurred at a very early stage: at 3 h of treatment, p-Rad17 was enhanced by 2.2-fold and then increased to a peak level of 4.3-fold of the controls at 6 h of exposure, which was consistent with the results from the protein array (Figure 2). TNFR1 expression was also elevated at 3 h post-treatment and reached the maximum of 4.1-fold at 12 h of exposure time.

TNFR1 induction by atrazine occurred at an early stage of atrazine treatment, which started to increase at 3 h post-treatment and reached its peak at 12 h (Figure 3B). Its mRNA was upregulated as early as 1.5 h by real-time PCR (data not shown). Atrazine-induced TNFR1 may suggest that TNF signaling is activated and that extensive downstream biochemical events are initiated by atrazine. The activated TNF signal could induce apoptosis in the cells, especially at higher concentrations. Liu *et al.* [33] showed that intracellular Ca^2+^, reactive oxygen species (ROS), mitochondrial membrane potential and ATP generation play important roles in atrazine-induced apoptosis in carp ZC7901 cells. However, in our study, protein array analysis showed that some pro-apoptosis proteins in the mitochondrial pathway were actually inhibited by atrazine, including Bad and Bcl-2 (Figure 2). This discrepancy may result from a much lower atrazine level and the different cell types that we used or some genes may be modulated by atrazine without statistical significance, but could result in biological events, including apoptosis.

### 2.4. Activation of ATR-Chk1 Pathway

**Figure 4:**
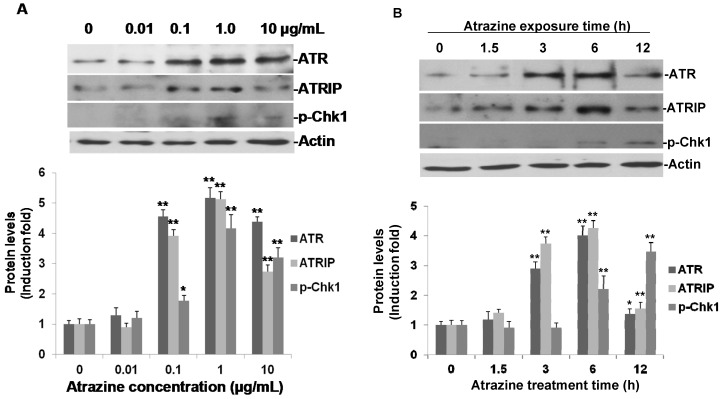
Atrazine-induced expression of ATR, ATRIP and phosphorylation of Chk1 (Ser317) in MCF-10A cells. (**A**) Dose-response of atrazine–induced ATR, ATRIP and p-Chk1. MCF-10A cells (3 × 10^5^) were incubated with the indicated concentrations of atrazine for 6 h and analyzed by Western blot; (**B**) time course of atrazine-induced ATR, ATRIP and p-Chk1in MCF-10A cells. MCF-10A cells (3 × 10^5^) were treated with 0.1 μg/mL of atrazine for the indicated times and analyzed by Western blot. For each blot, the resulting fold-change of atrazine-mediated ATR, ATRIP and p-Chk1 expression over DMSO untreated controls is provided in the corresponding histogram (*n* = 3) with the signal normalized to actin as a loading control. * *p* < 0.05, ** *p* < 0.01 *vs.* controls.

As p-Rad17 is one of the central DNA damage checking point proteins, we presumed that atrazine could activate the DNA damage response ATR-Chk1 pathway. To address that point, Western blot analysis was performed using specific antibodies against ATR, ATRIP and phospho-Chk1 (Ser317). As expected, dose-response experiments showed that ATR, ATRIP and phospho-Chk1 were induced greatly by treatment of atrazine for 6 h (Figure 4). Atrazine did not increase ATR, ATRIP and phpspho-Chk1 expression at a concentration of 0.01 µg/mL (Figure 4A). However, at higher concentrations (0.1, 1.0, and 10 µg/mL), atrazine obviously elevated all three protein levels, and the induced proteins reached their peak levels at a concentration of 1.0 µg/mL. At a concentration of 0.1 μg/mL, atrazine induced 4.5-, 3.9- and 1.7-fold of ATR, ATRIP and p-Chk1, respectively. We used a 0.1 μg/mL concentration of atrazine for time-course experiments, and we observed that ATR and ATRIP were induced 3 h post-treatment; and like p-Rad17, both ATR and ATRIP reached their high peaks at 6 h of treatment and then reverted to the baseline by 12 h of treatment (Figure 4B). Atrazine induced 4.0- and 4.3-fold of ATR and ATRIP at 6 h of treatment time compared with untreated controls (Figure 4B). There was no noticeable change on p-Chk1 at 3 h of treatment time, but an elevated p-Chk1 was observed at both of 6 (2.2-fold of controls) and 12 h of treatment (3.5-fold of controls). Our data further strongly suggested that the DNA damage response ATR-Chk1 pathway was triggered by atrazine in MCF-10A cells at an environment-relative concentration of 0.1 μg/mL.

ATR responds to a wide variety of DNA damage architectures, including UV-induced base damage, replication stress and DNA DSBs [34]. ATR phosphorylates and activates the Chk1 kinase in response to genotoxic stress [35]. Chk1 then proceeds to phosphorylate a variety of proteins that regulate aspects of the DDR, including cell cycle arrest, stabilization of stalled replication forks and DNA repair [32]. As such, the ATR-Chk1 axis is central to the DDR and crucial for maintaining genome integrity. Currently, the best measure of ATR activation is phosphorylation of CHK1 at the ATR-dependent residues Ser317 and Ser345 [36]. Our data revealed that atrazine indeed increased ATR and ATRIP (Figure 4A,B). Atrazine also activated Chk1 through phosphorylation, but from our Western blotting data, the obvious elevation was observed a little later than the activation of ATR and ATRIP (Figure 4B). That may due to the lesser amount of phosphorylation of protein Chk1 at 3 h of treatment time, leading to phospho-Chk1 being undetectable by Western blotting, or may suggest the sequential phosphorylation of these DNA damage checkpoint proteins. It is noticeable that p-Rad17, ATR, ATRIP and p-Chk1 decreased with the highest concentration of atrazine treatment (Figure 4B); this may result from the high concentration of atrazine that induces apoptosis in MCF-10A cells, as highly elevated TNFR1 was observed in the cells treated with 10 μg/mL of atrazine for 6 h of treatment time (Figure 4A).

### 2.5. Induction of γH2AX and γH2AX Foci

**Figure 5:**
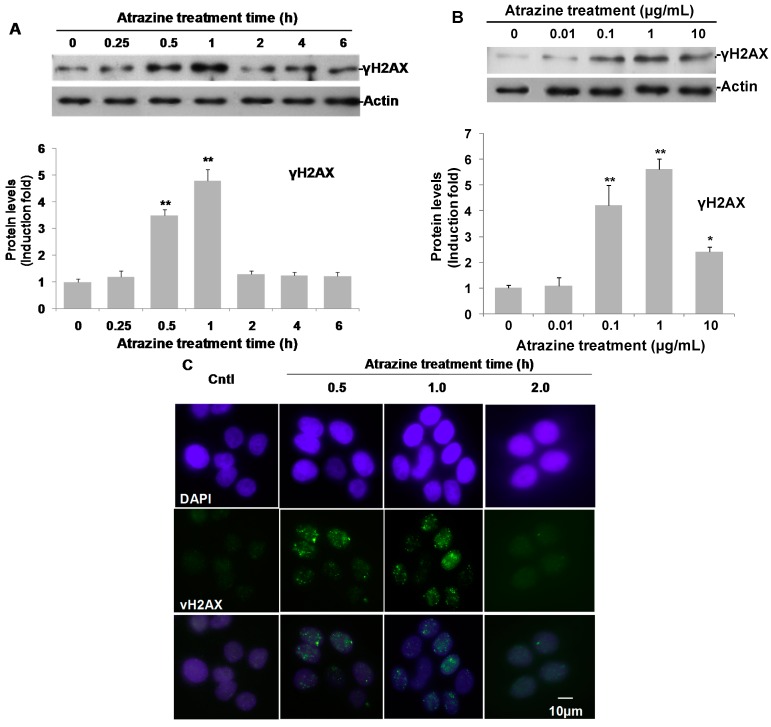
The induction of γH2AX in MCF-10A cells by atrazine. (**A**) Time course of atrazine-induced γH2AX. MCF-10A cells (3 × 10^5^) treated with 0.1 μg/mL atrazine for indicated hours and analyzed by Western blotting; (**B**) dose-response of atrazine-induced γH2AX. MCF-10A cells (3 × 10^5^) were exposed to the indicated concentrations of atrazine for 1 h and analyzed by Western blotting. For each blot, the resulting fold-change of atrazine-mediated phospho-H2AX (Ser139) expression over DMSO untreated controls is provided in the corresponding histogram (*n* = 3) with the signal normalized to actin as a loading control; (**C**) The representative images showing γH2AX foci induced by 0.1 μg/mL of atrazine for indicated hours. Phospho-H2AX antibody was indirectly labeled with Alexa Fluor 488 secondary antibody (green), and cells were mounted with VECTASHIELD Mounting Medium with DAPI (purple). The images were captured using a Carl Zeiss confocal microscopy using the same exposure time. * *p* < 0.05, ** *p* < 0.01 *vs.* controls.

The DNA damage response ATR-CHK1 pathway is activated when the cells are under genotoxic stress [35]. To further investigate the DNA damage induced by atrazine in MCF-10A cells, DNA double-strand breaks were examined by detecting phosphorylated H2AX (Ser139) and the foci of γH2AX (phosphorylated H2AX (Ser139)), which is the best tool available to examine the double-strand breaks in cells [37]. It was suggested that both the onset of DNA damage and repair occurred quickly after the cells were exposed to genotoxic stress. For example, γ-radiation-induced DNA double-strand breaks in human cells can be observed with γH2AX foci as early as 15 min after treatment, and γH2AX reverted to the baseline level by 4 h of exposure due to DNA repair [38]. Thus, in this study, γH2AX kinetics was monitored from a 15-min to 6-h span in response to 0.1 μg/mL of atrazine treatment. A slight increase of γH2AX was detected at 15 min. After 30 min and 1 h of treatment, γH2AX significantly increased to 3.6- and 4.7-fold of the controls respectively, as evaluated by Western blotting (Figure 5A). Dose-response experiments also showed that γH2AX was induced by atrazine at concentrations from 0.1 to 10 µg/mL when treating the cells with atrazine for 1 h (Figure 5B). Atrazine-induced γH2AX reached to a peak level at 1.0 µg/mL, which was 5.7-fold of the untreated controls (Figure 5B).

Immunofluorescence imaging further strengthened our findings in Western blot analysis (Figure 5A,B). After treatment with 0.1 μg/mL of arazine for 30 min and 1 h, γH2AX foci visualized as bright spots in confocal images were significantly induced (Figure 5C). In untreated cells, no obvious γH2AX foci were observed. However, after treatment with 0.1 μg/mL of atrazine for 30 min, γH2AX foci clearly appeared and reached peak levels at 1 h of treatment time (Figure 5C). Both γH2AX protein and γH2AX foci declined noticeably and returned to baseline levels in the treated cells 2 h post-treatment, suggesting that atrazine induced a burst of the DSBs followed by rapid DNA damage repair in MCF-10A cells (Figure 5A,C). Our data selectively suggested that atrazine at a concentration of 0.1 μg/mL could induce double-strand DNA damage in MCF-10A cells.

Although p-RAD17, ATR, p-Chk1 and γH2AX were induced by atrazine in our study, activation of another tumor suppressor and important cell cycle checkpoint protein p53 was not detected in the apoptosis antibody array (Figure 2). This could be due to the fact that the low level of phospho-p53 activated by atrazine could not be detected by the protein array or, most likely, atrazine induces DDR activation in a p53-independent fashion, as DDR activation could be p53-independent [39]. Previous studies on the genotoxicity indicated only minimal or no effect of atrazine on DNA integrity using the comet assay and micronuclei assay [40,41]. However, those methods may not be sensitive enough to detect low amounts of DNA damage. γH2AX is regarded as the most sensitive monitor currently available for high-throughput screening of genotoxic compounds [42]. H2AX, a minor histone H2A variant, plays a vital role in nucleosome formation in chromatin and is the earliest target in DNA damage [43]. H2AX would become phosphorylated (γH2AX) on serine 139 by ATM, ATR and DNA-PKcs as a reaction on DNA DSB. In this study, both immunoblotting and immunofluorescence imaging detected γH2AX induced by atrazine (Figure 5B,C).

DNA DSBs are the most harmful initial event in cell carcinogenesis [44]. Efficient DSB repair is crucial for the maintenance of genomic integrity. The DNA DSBs observed in our study may be due to the effects of ROS induced by atrazine, as studies showed oxidative damage of atrazine in various cell lines [45,46]. Furthermore, γH2AX levels were decreased after 2 h of treatment of atrazine (Figure 5A,C). That may suggest that atrazine-induced DNA damage does not have very important biological consequences due to the DNA repair and metabolism *in vivo*. However, although the DSBs induced by atrazine are transient and can be efficiently repaired in MCF-10A cells, its potential genotoxicity should not be neglected due to the fact that DSB can be repaired and we did not assume that the repair was not genotoxic. In human cells, the major DSB repair pathway (NHEJ) is highly error prone. Furthermore, atrazine is not used as a pure active ingredient in application, but in the form of commercial formulations. These formulations contain substances or compounds that might interact with atrazine and elevate its adverse effects. For example, the commercial formula Gesaprim^®^ and Gesaprim^®^ adjuvant mixture and their metabolites have been shown to cause DNA damage with a comet and DNA diffusion assay [47]. Atrazine was also found to enhance arsenic toxicity in human cells [48] and enhances oncogene expression of ANP32A in human breast epithelial cells [49]. As γH2AX has recently been used as an environmental monitor of genotoxic compounds [42], our data clearly show that atrazine can induce DNA damage in MCF-10A cells at environment-related levels.

## 3. Experimental Section

### 3.1. Chemicals and Antibodies

Atrazine (98% purity) from Superlco (Catalog No. 49085, Bellefonte, PA, USA) was dissolved in 100% DMSO to make a stock solution of 10, 1.0, 0.1 and 0.01 mg/mL. The CellTiter 96^®^ Aqueous One Solution Cell Proliferation Assay (MTS) kit was from Promega Company (Catalog No. G3580, Madison, WI, USA). The Human Apoptosis Array kit (Catalog No. ARY009) was from R&D Systems (Minneapolis, MN, USA). The Pierce BCA protein assay kit (Product No. 23227) and DAPI (Catalog No. H-1200) were from Thermal Fisher Scientific (Waltham, MA, USA).

Specific antibody against TNFR1 (Catalog No. 3736), phospho-Chk1 (Catalog No. 2344p) and ATRIP (Catalog No. 2737s) were purchased from Cell Signaling Technology (Danvers, MA, USA). Phospho-hRad17 (Ser635) (Catalog No. AF1374) was from R&D System, Inc. ATR (Catalog No. PA1-450) was from Thermo Fisher Scientific, and phospho-H2AX (Catalog No. 05-636) was from EMD Millipore (Darmstadt, Germany). Secondary antibodies for immunofluorescence imaging of p-H2AX were from Life Technologies (Catalog No. 21200, Grand Island, NY, USA).

### 3.2. Cells and Cell Culture

MCF-10A cells from American Type Culture Collection (Catalog No. CRL-10318, Manassas, VA, USA) were grown as a monolayer in TC175 flasks at 37 °C in a humidified incubator supplied with 5% CO_2_. The cells were maintained in Mammary Epithelium Basal Medium (MEGM) (Lonza, Walkersville, MD, USA) added with 10 ng/mL hEGF, 5 µg/mL insulin, 0.5 µg/mL hydrocortisone gentamicin and amphotericin-B. Before use, the medium was completed with bovine pit extract (BPE) (Catalog No. CC-4009, Lonza, Walkersville, MD, USA) at a final concentration of 0.4%. Cells were passaged twice a week. When treating the cells with atrazine, 1.0 μL of 10, 1.0, 0.1 and 0.01 mg/mL of atrazine stock solution was added into 1.0 mL of complete MEGM medium to prepare 10-, 1.0-, 0.1- and 0.01-µg/mL final treatment concentrations. An equal volume of DMSO was added as a vehicle control.

### 3.3. Cell Proliferation and Toxicity Assay

Cell proliferation and cytotoxicity were measured using a CellTiter 96^®^ Aqueous One Solution Cell Proliferation Assay (MTS) kit, according to the manufacturer’s instruction. Briefly, ninety-six well plates were seeded with 10,000 cells (per well) in 200 µL of completed MGEM medium. After cells were allowed to attach to the surface overnight at 37 °C, as described above, the medium was carefully removed. Medium with different concentrations of atrazine or an equal volume of DMSO was added into the wells and incubated for 6, 12, 24 and 48 h. At the end of incubation, 20 μL of the cell proliferation reagent (MTS) were added to the medium and incubated for another 1.5 h at 37 °C. The absorbance of the treated samples against a blank control was measured using a microplate reader (SYNERGY 4, Biotek, Winooski, VT, USA) with a test wavelength of 450 nm and a reference wavelength of 690 nm. Cell proliferation was performed in 4 wells, and each experiment was triplicated.

### 3.4. Human Apoptosis Antibody Array

The expression profiles of 35 apoptosis-related proteins were detected and analyzed using a human apoptosis array kit following the manufacturer’s procedure. Briefly, MCF-10A cells (1 × 10^6^) were seeded in 60 mm × 15 mm sterile dishes in 6 mL of complete medium and incubated overnight at 37 °C, then treated with or without atrazine (0.1 µg/mL) for 6 h. The cells were harvested, and the cell pellets were lysed in 100 µL lysis buffer (8 M urea, 50 mM Tris, pH 7.5, 150 mM 2-mecaptoethanol) and centrifuged at 16,000× *g* for 15 min at 4 °C. The supernatant were collected and kept at −80 °C until use or directly applied to the human apoptosis antibody array assay. The nitrocellulose membranes containing immobilized specific monoclonal antibodies against 35 apoptosis-related proteins were blocked with bovine serum albumin for 1 h on a rocking platform at room temperature. The membrane was then incubated with supernatant lysates of MCF-10A cells treated with or without atrazine (0.1 µg/mL) overnight at 4 °C on a rocking platform. After 3 washes (each wash for 5 min) of the membranes with 2 mL of wash buffer, the membranes were then incubated with the detection antibody cocktail for 1 h on a rocking platform, followed by 3 washes. Then, the membranes were incubated with streptavidin-horseradish peroxidase conjugate and by a chemiluminescent detection reagent. The membrane was then scanned using a G:Box Chemi (Syngene, Frederick, MD, USA), and pixel density was presented by quantifying the mean spot densities from three replicates.

### 3.5. Western Blot Analysis

MCF-10A cells (3 × 10^5^) were seeded into a 6-well plate in 2 mL of complete culture medium and incubated overnight, then treated with atrazine. For time course experiments, the cells were treated with 0.1 μg/mL of atrazine for the indicated hours. For dose-response experiments, the cells were treated with indicated concentrations of atrazine for 6 h. Before harvest, the cells were washed with 5 mL of PBS twice, then lysed in 150 µL of lysis buffer (per well) containing 50 mM Tris (pH 7.4), 1% Triton X-100, 1% Nonidet P-40, 150 mM NaCl, 0.4 mM EDTA, 0.2 mM EGTA, 0.2 mM vanadate and 1% protease inhibitor cocktail (Catalog No. 87786, Thermo Scientific, Rockford, IL, USA). The lysates were centrifuged at 16,000× *g* for 15 min at 4 °C, and the supernatants were measured for protein concentration using a Pierce BCA protein assay kit. A total protein of 20 μg per sample was separated in 12% SDS-PAGE gel and transferred to PVDF membranes (Catalog No. IPVH00010, Millipore, Billerica, MA, USA) by electrotransfer. The blots were subsequently blocked with 5% non-fat milk powder in TBS containing 0.1% Tween 20 (TBS-T) at room temperature for 1 h and incubated at 4 °C overnight with primary antibodies of interest. After washing three times for 10 min with TBS-T, the membrane was incubated with horseradish-peroxidase-linked secondary antibodies of interest at room temperature for 1 h. The blots were visualized with the ECL Western blotting substrate (Catalog No. 32106, Thermo Scientific, Rockford, IL, USA) and imaged using a FluorChem M digital imager (ProteinSimple, Santa Clara, CA, USA). The same membrane was re-probed with an anti-human actin antibody as an internal loading control. Band density on Western blot images was used as a measure of assayed protein level and analyzed by the AlphaView SA software.

### 3.6. Immunofluorescence Imaging of γH2AX Foci

Cover slips coated with 40 µg/mL of poly-l-lysine were put in 35-mm petri dishes, then MCF-10A cells (1 × 10^6^) were seeded into the petri dishes in 6 mL of complete MEGM medium. After 24 h of culturing to allow the cells to attach to the slips, cells were incubated with or without 0.1 µg/mL of atrazine for 0.5, 1 and 2 h, and then, cell layers in the slips were washed 3 times with 2 mL PBS and fixed in 4% paraformaldehyde for 20 min at room temperature. Afterward, the cell layers were washed twice with 2 mL of PBS. For immunofluorescence staining, the cells were permeabilized for 3 min in 0.25% Triton X-100 in PBS, washed twice by 2 mL of PBS and blocked for 1 h with 5% BSA in PBS. The cells were incubated at 4 °C overnight with primary antibodies against γH2AX (Ser139) at a 1:200 dilution, washed three times with PBS, followed by incubation with secondary antibodies of interest at a 1:400 dilution for 2 h at room temperature. The slides for γH2AX were mounted on cover slides with DAPI. Images were captured using a Carl Zeiss confocal microscopy.

### 3.7. Statistics

All data are expressed as the mean ± SD (standard deviation). Differences among treatments were assessed by the one-way analysis of variance (ANOVA). If the variances among treatments were homogenous, data were subjected to the multiple comparison Dunnett’s test using a 5% significance level (*p* < 0.05).

## 4. Conclusions

In conclusion, this research presented the first study to investigate the genotoxicity of atrazine at environmentally-detectable levels in human MCF-10A cells. The results demonstrated that atrazine not only increases the expression of death receptor TNFR1 and phosphorylated Rad17 (Ser635), but also induces the phosphorylation of H2AX and γH2AX foci. Atrazine at environmentally-detectable levels could trigger the DNA damage response ATR-CHK1 pathway. With highly sensitive assays, this study provided a direct molecular evidence of the most widely-used atrazine for its genotoxicity and possible carcinogenic properties in human mammary cells. Further investigations are needed to determine whether the atrazine-triggered DNA double-strand breaks and DNA damage response ATR-Chk1 pathway occur *in vivo*.
